# Subjective memory and 5-year all-cause mortality in subjective cognitive decline, non-amnestic mild cognitive impairment, and amnestic mild cognitive impairment

**DOI:** 10.1007/s11357-025-01993-z

**Published:** 2025-11-11

**Authors:** Paula A. Grässler, Johann Lehrner

**Affiliations:** Department of Neurology, Medical University of Vienna, Währinger Gürtel 18-20, Vienna, 1090 Austria

**Keywords:** Subjective memory, 5-year mortality, SCD, NaMCI, AMCI, Neurocognitive function

## Abstract

Cognitive decline is becoming increasingly prevalent, and dementia diseases are currently the seventh leading cause of death worldwide—the trend is rising. Consequently, it is imperative to identify influencing factors on the mortality of cognitively impaired patients. So, the main aim of this study was to determine the impact of subjective memory on 5-year mortality in Subjective Cognitive Decline (SCD), non-amnestic Mild Cognitive Impairment (naMCI), and amnestic Mild Cognitive Impairment (aMCI). A Cox proportional hazard model was applied to assess whether subjective memory performance (FAI) predicts mortality across SCD, naMCI, and aMCI. It was based on a university-based single-center retrospective data analysis with *n* = 1004 adjusted patient records from the Department of Neurology of the Medical University of Vienna over the observation period 2000–2022. Covariates such as sociodemographic factors (age, gender, years of schooling), depression (BDI-II), and neuropsychological performance (NTBV) were included blockwise. Study findings suggest that subjective memory performance could not be identified as an explanatory value for mortality in the study population, *p*’s ≥ .181. Rather, increasing age (HR 1.083, *p* < .001) and male gender (inverted HR 1.517, *p* = .004) were identified as relative risk factors. Also, four NTBV dimensions emerged as significant factors: decreased attention (HR 1.417, *p* < .001), diminished executive function (HR 1.283, *p* < .001), reduced objective memory (inverted HR 1.253, *p* = .001), and a declined language (inverted HR 1.166, *p* = .026) can be assumed to be relative risks for higher mortality.

## Introduction

Globally, dementia affects more than 55 million individuals, with projections estimating a rise to 78 million by the year 2030. Moreover, the World Alzheimer’s Report 2021 determined that dementia is the seventh leading cause of death worldwide [1].

Dementia-related diseases are mostly associated with impaired memory, behavior, thinking, and social functioning. Additional symptoms may present depending on etiology and individual factors, including aggression, aphasia, blindness, or loss of orientation [1].

Cognitive impairments, both subjective and objective, can be prevalent even in the absence of a formal dementia diagnosis [2]. Previous studies have investigated potential factors that may influence mortality in these patients. The findings indicate that elevated HbA1c levels are associated with a significant increase in 5-year mortality [3]. However, no studies could be found that examined the influence of subjective memory on mortality in cognitively impaired patients.

Subjective memory is known as the personal perception of an individual’s own memory performance, particularly in comparison to one’s younger self and within an age-matched environment [4]. Research findings indicate the presence of potential associations with objectively verifiable memory performance. However, these associations are not always detectable and tend to be low [5, 6].

Patients who report a persistent subjective decline in cognition despite normal objective neuropsychological test results, thus not meeting criteria for MCI or dementia, may be diagnosed with SCD. The perceived impairment must reflect a change from usual cognitive functioning and cannot be attributed to acute events [7–9]. Symptoms can occur in different cognitive domains, depending on the underlying condition. Memory, processing speed, visuospatial abilities, and executive functions are particularly affected [10].

SCD has been associated with a variety of factors, including normal aging processes, female gender, less education [11], psychiatric, neurological, or internal diseases, infections, nutritional deficiencies, use of substances or medication, and cultural background [9, 12]. A meta-analysis by Mitchel et al. found that the probability of developing dementia in patients with SCD is twice that of the age-matched cognitively unimpaired normal population [13]. Also, depression is closely interwoven with subjective memory loss. Depression may imitate cognitive decline [14] while, conversely, SCD can cause depressive symptoms [4]. Furthermore, both conditions may be accompanied by a loss of awareness [15]. Depressiveness can also reflect early neurodegenerative changes. Recent evidence indicates that it can occur years before measurable cognitive decline or awareness deficits [16]. So, depression, SCD, and MCI can each be considered risk factors for each other [17, 18].

In contrast to SCD, people with MCI are conspicuous in objective neuropsychological tests. MCI can be subclassified based on the affected cognitive domains. When memory function is primarily affected, the condition is defined as amnestic MCI (aMCI). Typically, episodic memory declines, while basic activities of daily living remain unaltered. This distinguishes it from cases of dementia [19–21]. Conversely, if impairments are observed in non-memory domains, such as executive function, language, or visuospatial processing, the condition is referred to as non-amnestic MCI (naMCI) [21, 22].

Studies indicate that individuals with MCI exhibit an elevated risk of progressing to dementia later in life than people in the age-matched general population [23]. Especially aMCI often represents a prodromal stage of Alzheimer’s dementia, whereas naMCI may result from cerebrovascular disease, frontotemporal degeneration, or other causes, such as other neurologic, psychiatric, or systemic ones [24, 25]. Cognitive deficits due to cerebrovascular pathology are also known as vascular cognitive impairment (VCI) [26].

The prevalence of self-observed memory problems among individuals over 65 varies across studies, ranging from 12.3 to 57% due to variations in the diagnostic instruments employed and the specific assessments [5, 11, 18, 27]. It increases with age, reaching up to 88% in individuals aged 85 years and older [2]. The prevalence of MCI in people over the age of 60 is approximately 6%, also increasing with age, as evidenced by the findings of the COSMIC collaboration [28]. Conversely, 62% of patients with MCI and 60% of those with dementia deny experiencing subjective memory problems [18, 29], which is likely related to their loss of awareness of change [30].

The extant evidence regarding the association between SCD and an increased mortality risk is inconclusive. While some studies found no significant association [31–34], others reported an increased mortality among individuals with SCD [35, 36]. For instance, Luck et al. showed a 51% elevated risk in patients with SCD compared to the reference group [35]. Singh-Manoux et al. further suggested that this risk may vary depending on the affected cognitive domain, identifying mental calculation difficulties as a main risk factor [36].

Comparable inconsistencies have been observed in the context of MCI. A variety of studies have demonstrated that patients diagnosed with MCI exhibit a higher mortality rate compared to the age-matched cognitively normal population. For instance, Vassilaki et al. reported a mortality rate of 38.4% in the MCI cohort versus 17.3% in the cognitively unimpaired population over a median follow-up time of 5.8 years [37]. Other studies indicated a heightened risk associated with naMCI compared to aMCI [38–41]. Concurrently, Li et al. identified a 40% elevated mortality among Chinese individuals diagnosed with MCI. Their findings indicate that both diminished baseline cognitive function and rapid cognitive decline during the observation period were independently associated with heightened all-cause mortality [42]. However, certain investigations have been unable to identify a definitive association between MCI and increased mortality [3, 43–45].

The goal of the present study was to investigate the association between subjective memory function and the 5-year mortality in patients with SCD, naMCI, and aMCI.

## Methods

### Study design

This study is a university-based, single-center retrospective data analysis. The data were collected between March 2000 and December 2022 at the Department of Neurology at the Medical University of Vienna.

Data on the potential death of study participants was obtained from the Research, Documentation, and Analysis system of the Medical University of Vienna. Without a present death certificate on 17.12.2021, the patients were declared alive, as no data was available from Statistics Austria for the subsequent period at the time of the analysis.

### Participants

The participants were patients at the Department of Neurology at the Medical University of Vienna between March 2000 and December 2022. They all underwent a full physical and neurological examination and were diagnosed with one of three disorders: SCD, naMCI, or aMCI. Patients with SCD had been diagnosed by the criteria of Jessen et al. [9].To detect an MCI and distinguish the non-amnestic and amnestic types, the diagnosis criteria of Petersen were applied [23]. Additionally, sociodemographic factors such as age, gender, and educational status were ascertained. The participants also underwent neuropsychological testing.

Initially, a pool of 1175 participants, including 204 diagnosed with SCD, 549 with naMCI, and 417 participants with aMCI, was available. Participants were included at an age higher than 50 years, and when neuropsychological testing and the FAI were carried out on the same day. Patients with neurological diseases, manifest dementia, evidence of stroke, traumatic head injury in the past, pseudo-dementia caused by psychiatric problems [excluding (sub-)depressive symptoms], systemic medical conditions leading to cognitive impairment, and participants with incomplete data were excluded.

By applying the inclusion and exclusion criteria, 71 (6.0%) protocols had to be removed so that the final study collective comprises 1104 cases.

### Instruments

#### Subjective memory

The Forgetfulness Assessment Inventory (FAI) was used for quantifying the subjective memory function of the participants. The questionnaire has been validated for participants diagnosed with MCI [46, 47]. It contains 16 questions developed for detecting self-perceived problems in everyday memory within the past 4 weeks. The patients should answer between (1) never and (5) very often. The higher the score, the worse the subjective memory. The FAI can be obtained from www.psimistri.com [30, 48].

#### Neuropsychological functions

Neuropsychological tests were used to assess the cognitive status of the participants.

The Mini-Mental State Examination (MMSE) [49] and the VVT 3.0 were used to screen patients’ cognitive performance [50]. The VVT 3.0 can be obtained from www.psimistri.com [51].

Verbal intelligence and age-independent language comprehension were assessed using the Wortschatztest-IQ (WST-IQ). It can be considered an adequate indicator of a person’s general intelligence, as the number of words a person has can provide a measure of their stock of linguistic knowledge, learning ability, and general imagination [52].

The neuropsychological test battery Vienna (NTBV) is a series of neuropsychological tests that can be used to diagnose objective cognitive impairment and dementia. It includes different categories of cognitive performance to be investigated, which are characteristically impaired in Alzheimer’s dementia. These involve attention, executive function, language, memory, and psychomotor speed. In the present study, 24 subtests were performed and categorized into six cognitive domains. The NTBV can be obtained from www.psimistri.com [53].

#### Depressive symptoms

Furthermore, the BDI-II was performed. It is an assessment inventory to specify the severity of depressive symptoms in patients. It is designed in such a way that there are four statements for each of 21 symptoms, from which the test taker is to select the one that best fits his or her situation within the last 2 weeks. In total, this allows patients to score up to 63 points. Starting at 9 points, the patients are declared to have minimal depressive symptoms; if they get more than 29 points, it is already a manifested depression [54].

### Statistical analyses

Statistical analyses were performed using the IBM SPSS® 29.0.0 (IBM Corp, Armonk, NY, USA). The level of significance, corresponding to the type I error, was set at *α* = 5%, and all statistical procedures were conducted using a two-tailed approach. In cases of multiple testing, a Bonferroni adjustment with ${\alpha }^{*}=\frac{\alpha }{k}$ was implemented to mitigate the risk of Type-I-Error accumulation [55].

For descriptive analyses, metric parameters were characterized using the mean (M), standard deviation (SD), min–max range, 95% confidence interval (CI), median (Md), and interquartile range (IQR), both for the entire study sample and each of the diagnostic groups. Nominal variables were analyzed using frequencies (*n*) and proportional values (%). Chi-square testing was applied to examine the relationship between categorical parameters [55].

In the inferential part of the analysis, differences between diagnostic groups in metric, normally distributed parameters (*n* ≥ 30; verified by Kolmogorov–Smirnov and Shapiro–Wilk tests) were examined using analysis of variance (ANOVA) [56]. Levene’s procedure was employed to test for homogeneity of variances, and Welch one-way ANOVA was applied in case of heterogeneity [57, 58]. For at least ordinally scaled parameters, group differences were analyzed using the Kruskal–Wallis test [59].

Post hoc comparisons were conducted using the Games-Howell procedure or Mann–Whitney *U* testing, and Welch’s *t*-test was applied when variances were unequal [57].

The Wilcoxon signed-rank test was employed for comparisons with a constant reference value.

Effect sizes were calculated according to Cohen’s classification for parametric tests (*d* ≥ 0.20 small, ≥ 0.50 moderate, ≥ 0.80 large) and for non-parametric tests using $r= \frac{z}{\sqrt{N}}$ (*r* ≥ 0.10 small, ≥ 0.30 moderate, ≥ 0.50 large) [56, 59, 60].

Survival rates across the diagnostic groups were estimated using the Kaplan–Meier method [61], with mortality differences assessed by the log-rank test [62]. Cox regression was applied to evaluate the impact of subjective memory on mortality. To control for confounders, other variables such as depressive symptoms, sex, age, school years, and neuropsychological abilities were added blockwise and hierarchically [63].

To reduce the dimensionality of the 24 NTBV subscales, a principal component analysis (PCA) with orthogonal rotation (Kaiser–Varimax approach) was performed. Sampling adequacy was evaluated using the Kaiser–Meyer–Olkin coefficient (KMO > 0.50) [64]. Communality and eigenvalue (*λ*) were considered key values, and *Z*-standardized factor scores were computed to obtain uncorrelated measures [65, 66].

## Results

### Demographic characteristics

The sociodemographic characteristics of the study population, as well as the results in cognitive and depressive symptoms testing regarding the diagnostic subgroups, are depicted in Table 1.

**Table 1:** Descriptive statistics (n, Md, IQR, frequencies, percentages) of the study-relevant parameters considering diagnostic subgroups and p-values from group comparisons

Parameter	Diagnostic subgroup			Total *N* = 1104	*p*
	SCD *n* = 200	naMCI *n* = 512	aMCI *n* = 392		
Sex Male	101 (50.5%)	191 (37.3%)	197 (50.3%)	489 (44.3%)	<.001
Female	99 (49.5%)	321 (62.7%)	195 (49.7%)	615 (55.7%)	
Age at testing	66.2 (59.4; 74.0)	67.6 (59.7; 74.4)	69.3 (61.3; 75.8)	68.1 (60.4; 74.8)	.038
Age at death	84.8 (77.5; 89.2)	83.0 (76.2; 89.5)	80.2 (74.2; 86.0)	82.2 (75.6; 88.6)	.057
Formal years of education	12 (9; 16)	11 (8; 15)	12 (8; 16)	12 (8; 16)	.334
FAI (1–5)	2.81 (2.25; 3.28)	2.88 (2.31; 3.38)	3.00 (2.50; 3.56)	2.88 (2.31; 3.38)	<.001
MMSE (0–30)	29 (28; 30)	28 (27; 29)	28 (26; 29)	28 (27; 29)	<.001
WST-IQ (*µ* = 100, *σ* = 15)	114 (107; 122)	110 (99; 118)	107 (97; 118)	110 (99; 119.5)	<.001
VVT 3.0 change (− 10–10)	− 1 (− 4; 0)	− 2 (− 5; 0)	− 5 (− 7; − 2)	− 3 (− 5; 0)	<.001
BDI-II (0–63)	8 (5; 14)	10 (5.5; 16)	9 (5; 15)	9 (5; 15)	.047
F1 Attention^1^	− 0.40 (− 0.69; − 0.02)	− 0.06 (− 0.63; 0.60)	− 0.09 (− 0.65; 0.71)	-	<.001
F2 Executive function^1^	− 0.29 (− 0.71; 0.30)	− 0.06 (− 0.67; 0.58)	0.75 (− 0.69; 0.65)	-	.003
F3 Memory	0.46 (− 0.05; 1.01)	0.49 (− 0.00; 0.99)	− 0.76 (− 1.35; − 0.26)	-	<.001
F4 Planning	0.26 (− 0.10; 0.83)	− 0.02 (− 0.71; 0.61)	− 0.03 (− 0.64; 0.68)	-	<.001
F5 Language	0.36 (− 0.08; 0.97)	− 0.10 (− 0.75; 0.56)	− 0.10 (− 0.85; 0.60)	-	<.001
F6 Nonverbal fluency^1^	− 0.31 (− 0.66; 0.33)	− 0.08 (− 0.61; 0.51)	− 0.17 (− 0.66; 0.44)	-	<.001

^1^Inversed, lower values indicating better performance

The study collective included 55.7% women, which did not significantly differ from the gender distribution in the Austrian population aged 50–90 years in 2021 (52.78% female [67]; *χ*^2^(1) = 3.786, *p* = 0.052). However, a significant discrepancy in the distribution of sex was observed across diagnostic subgroups (*χ*^2^(2) = 18.904, *p* < 0.001). The naMCI participants showed a notably higher proportion of women (62.7%, *z* = + 2.12 > *z*_crit._ |1.96|).

The mean age of the participants was 67.7 years. There was a significant difference in age between diagnostic groups (univariate two-way ANOVA; *p* = 0.038), with older age being associated with more severe cognitive impairment.

The subgroups did not vary significantly regarding their years of education (Kruskal–Wallis; *H* *χ*^2^(2) = 2.192, *p* = 0.334).

### Subjective memory

The FAI was used to quantify the self-reported memory function. Scoring between 1 and 5 points indicates self-estimated memory; the higher the result, the worse the subjective memory function appears. A significant difference showed up between the scoring of the diagnostic subgroups (one-way ANOVA; *F*(2, 1101) = 8.074, *p* < 0.001). So participants with SCD scored significantly lower than those with aMCI (Games-Howell; *p* < 0.001, *d* = 0.33) but not lower than those with naMCI (*p* = 0.244). However, the naMCI group did better than the aMCI group (*p* = 0.011, *d* = 0.19).

**Fig. 1:**
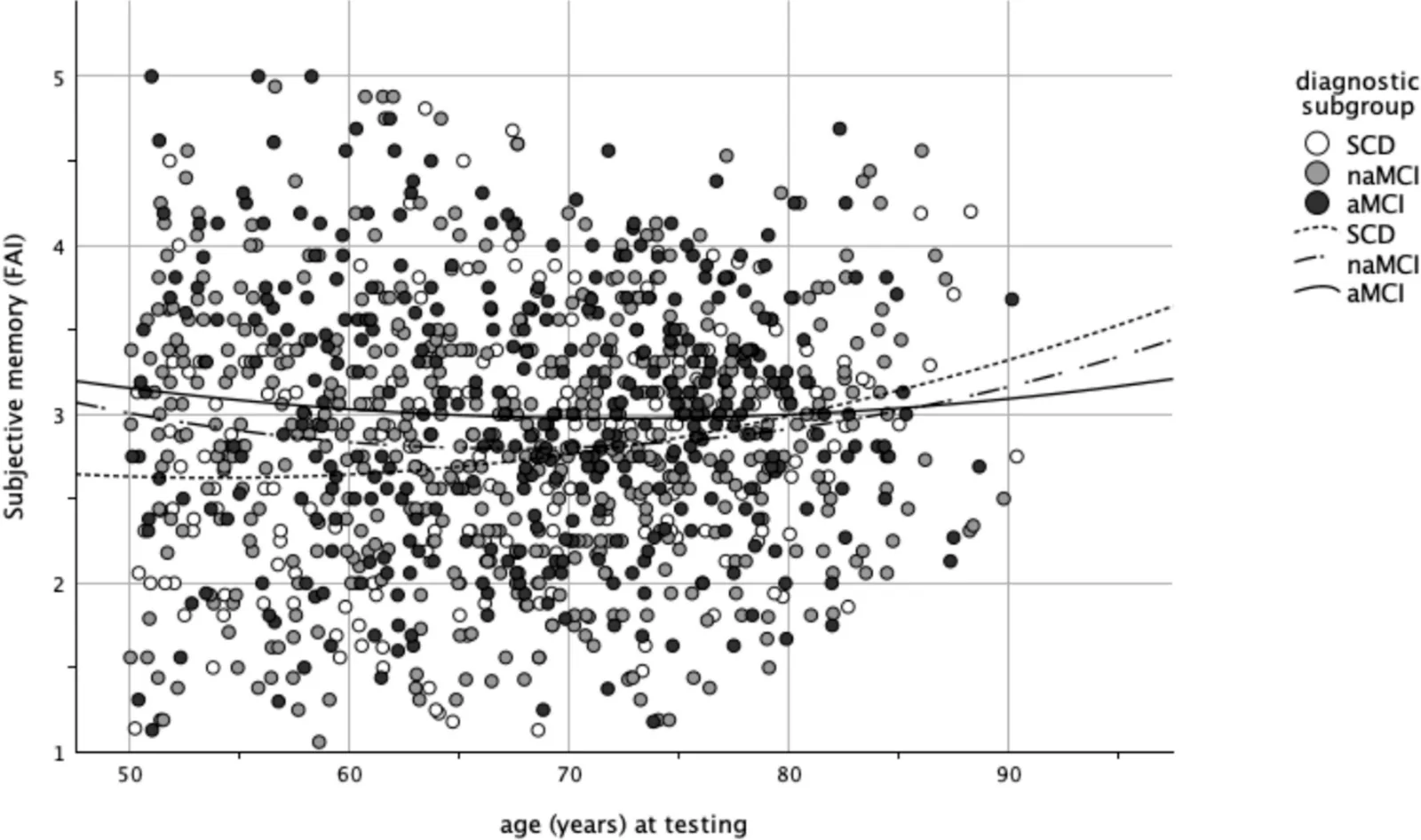
Bivariate scatterplot of the correlation between age (years) and FAI (score 1–5) considering diagnostic subgroups with quadratic regression functions (*n* = 1104)

Additionally, the relationship between age at testing and FAI was analyzed using Pearson’s correlation. It turned out that, taking the linear relation into account, participants with SCD subjectively rated their memory significantly worse with increasing age (*r* = 0.18, *p* = 0.010), while those with naMCI and aMCI showed a consistent, non-significant assessment (Fig. 1).

### Neuropsychological functions 

MMSE, WST-IQ, and VVT 3.0 were used for assessing patients’ cognitive performance. Inferential statistical analyses were used comparing results between the three diagnostic subgroups. Thereby a hierarchical performance gradient of SCD > naMCI > aMCI could be observed; results of inferential statistics are depicted at Table 2.

**Table 2:** Results of inferential statistical analyses comparing the scoring at the neuropsychological testings MMSE, WST-IQ, and VVT 3.0 between the diagnostic subgroups. The Kruskal–Wallis procedure and Mann–Whitney *U* tests were used for MMSE and VVT 3.0. Welch one-way ANOVA and Welch’s *t*-test were used for WST-IQ

Neuropsychological tests	Overall	SCD vs. naMCI	SCD vs. aMCI	naMCI vs. aMCI
MMSE	H *χ*^2^(2) = 96.195, ***p* <.001**	z = − 4.025, ***p* <.001**, *r* =.15	*z* = − 9.173, ***p* =.001**, *r* =.38	*z* = − 7.028, ***p* =.001**, *r* =.23
WST-IQ	*F*(2, 559.25) = 21.800, ***p* <.001**	*t*(410.89) = 5.311, ***p* <.001**, *d* = 0.42, 95% CI [0.25; 0.58]	*t*(453.12) = 6.397, ***p* <.001**, *d* = 0.53, 95% CI [0.36; 0.70]	*t*(837.64) = 1.617, *p* =.106
VVT 3.0	H *χ*^2^(2) = 16.624, ***p* <.001**	*z* = − 1.155, *p* =.248	*z* = − 3.725, ***p* <.001**, *r* =.36	*z* = − 3.332, ***p* <.001**, *r* =.25

The 24 NTBV-subtests were analyzed using a principal component analysis (PCA) with Varimax rotation (Kaiser’s normalization). Rotation converges after 12 iterations, yielding a six-factor solution Table 3. The KMO value (0.835) indicated suitability for factor analysis. Missing values were imputed using mean substitution.

**Table 3:**
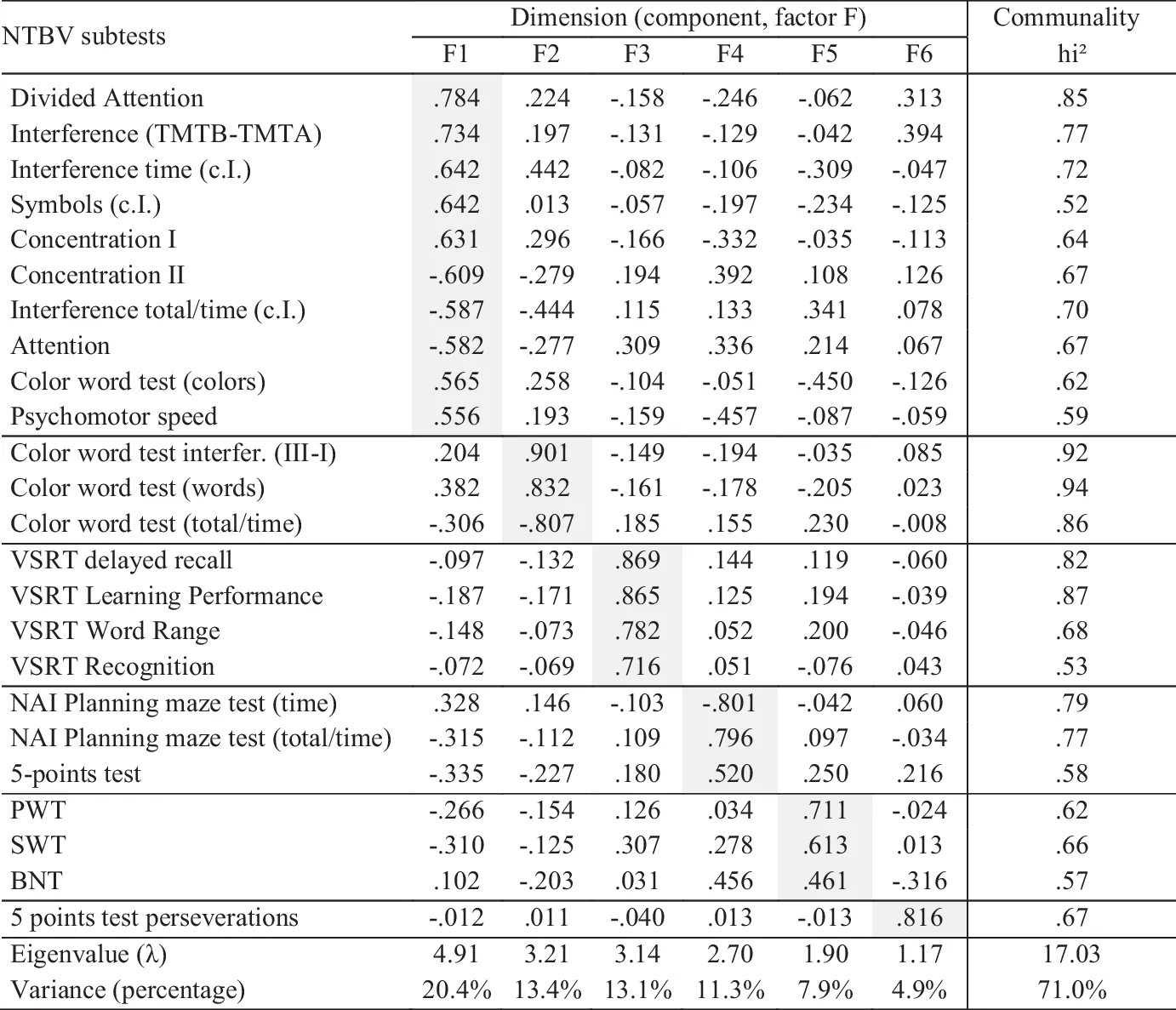
Rotated component matrix; factor loadings, communality, eigenvalue (*n* = 1104)

The extracted dimensions explained 71.0% of the cumulative variance. Dimension names were assigned based on highly loaded subtests, aligning with the original test battery’s domain categorization. Given NTBV’s mix of times, scores, and quotients, polarity was considered in interpretation. The six identified dimensions were F1—Attention, F2—Executive function (phonematic verbal fluency), F3—Memory, F4—Planning, F5—Language-verbal intelligence, F6—Nonverbal fluency.

To assess variations in the performance of the diagnostic groups across the six dimensions, the PCA-derived, weighted *z*-standardized factor scores were analyzed. Significant differences emerged in all dimensions (one-way Welch-ANOVAs; *p*_F1, 3–6_ < 0.001, *p*_F2_ = 0.003). SCD differed significantly from aMCI in all dimensions (Games-Howell; *p*_F1-6_ < 0.001) and from naMCI in all (*p*_F1, 4–6_ < 0.001, *p*_F2_ = 0.026) except the memory factor (*p*_F3_ = 0.003). NaMCI and aMCI groups, on the other hand, showed significant differences only in memory (*p*_F3_ < 0.001, *p*_F1_ = 0.703, *p*_2_ = 0.243, *p*_F4_ = 0.867, *p*_F5_ = 0.987, *p*_6_ = 0.684).

### Relation between objective and subjective memory performance

Participants’ objective memory ability (NTBV F3) and their self-esteemed performance (FAI) were, except for the naMCI group, significantly but weakly interrelated (Pearson’s correlation; *r*_total_ = −0.16, *p*_total_ < 0.001; *r*_SCD_ = −0.15, *p*_SCD_ = 0.030; *r*_naMCI_ = −0.081, *p*_naMCI_ = 0.068; *r*_aMCI_ = −0.134, *p*_aMCI_ = 0.008).

### Depressive symptoms

Depressive symptoms were surveyed using the BDI-II. The results of the subgroups differed significantly (Kruskal–Wallis; *H* *χ*^2^(2) = 6.135, *p* = 0.047). Participants diagnosed with SCD achieved a significantly lower extent than those with naMCI (Mann–Whitney *U*; *z* = −2.522, *p* = 0.012, *r* = 0.10). However, SCD vs. aMCI (*z* = −1.661, *p* = 0.097) and naMCI vs. aMCI (*z* = −0.823, *p* = 0.410), respectively, showed no significant differences.

### Mortality considering the diagnostic subgroups

The diagnostic subgroups did not differ significantly concerning their age of death, either among themselves (Kruskal–Wallis; *H* *χ*^2^(2) = 5.746, *p* = 0.057) or compared to the estimated age at death of the total Viennese population in 2023, which was 80.93 years [68] (Wilcoxon signed-rank test, *p*_total_ = 0.174, *p*_SCD_ = 0.090, *p*_naMCI_ = 0.085, *p*_aMCI_ = 0.499).

**Fig. 2:**
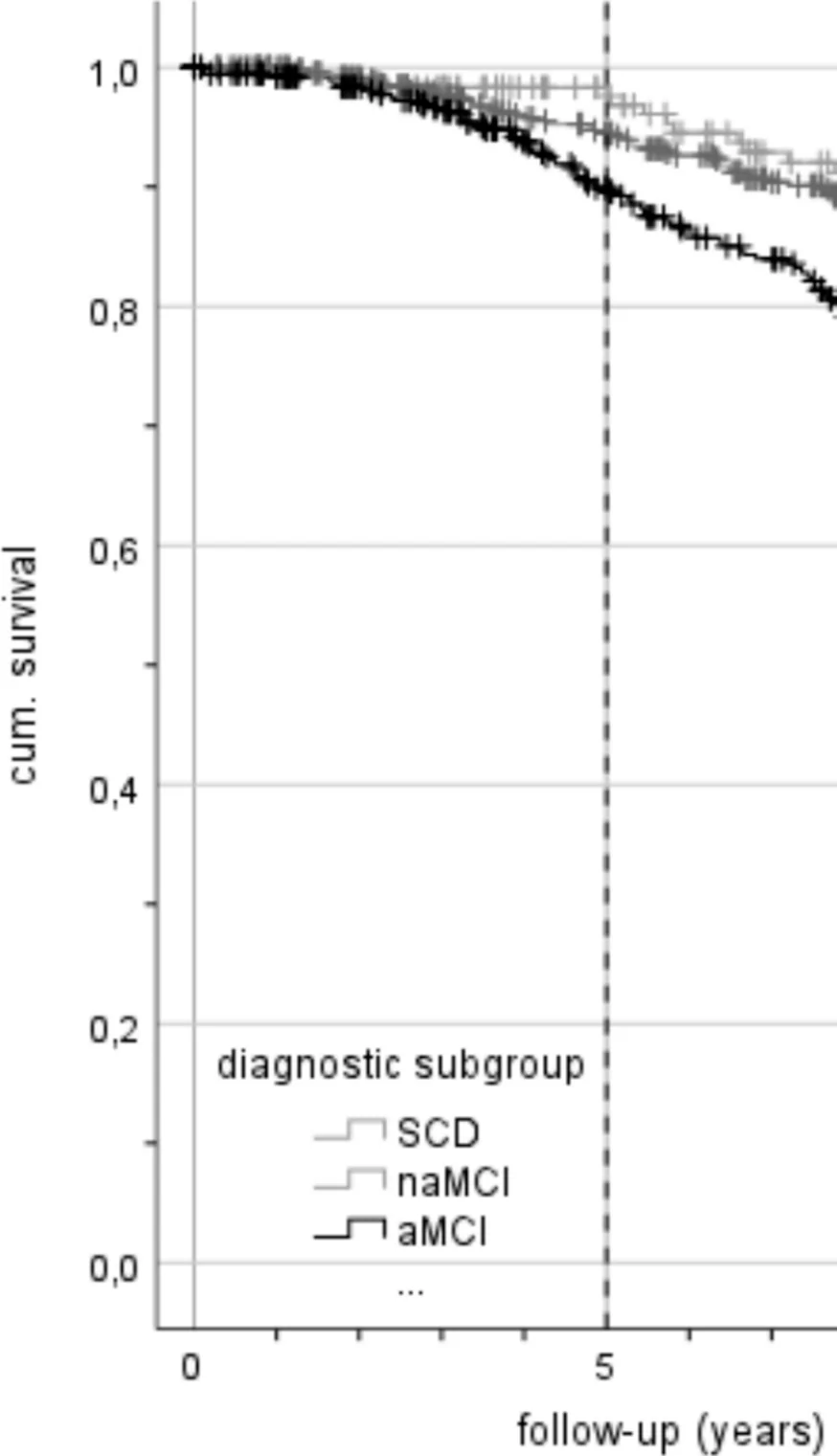
Kaplan–Meier survival functions considering diagnostic subgroups

The Kaplan–Meier procedure was employed to analyze the probability of survival considering the three diagnostic subgroups. The survival functions are depicted in Fig. 2.

The primary objective of the study was to examine the 5-year mortality rate of the participants. All three diagnostic subgroups differed significantly in this regard (log-rank testing; *p* = 0.004). Pairwise testing, taking Bonferroni adjustment (α* = 0.0167) into account, revealed no significant differences in survival between SCD vs. naMCI (*p* = 0.142), but between SCD vs. aMCI (*p* = 0.004), and naMCI vs. aMCI (*p* = 0.021).

In summary, it can be stated that the probability of survival for aMCI patients is already significantly lower 5 years after testing compared to patients with SCD or naMCI.

### Influencing factors on 5-year mortality

A multivariable Cox-proportional hazards model was employed to evaluate the explanatory value of predictors and covariates for mortality, as shown in Table 4. The model accounted for both censored and deceased cases by including follow-up time as a time-dependent variable. Predictors were entered hierarchically across five model blocks to evaluate changes in their effects. A total of 1059 cases were included in this analysis; 45 censored cases were excluded because their observation period was shorter than that of the first deceased case. Among the included cases, 264 (23.9%) had died, and 795 (72.0%) were censored.

**Table 4:** Coefficients of predictors considering the mortality criterion (*n* = 1059)

Predictor	*B*	SE	Wald *χ*^2^ (df = 1)	*p*-value	HR	95% CI HR	
						LL	UL
Block 1							
FAI	.035	.081	.189	.664	1.036	.884	1.213
Block 1–2							
FAI	−.032	.092	.119	.730	.969	.809	1.161
Age	.103	.008	169.410	<.001**	1.109	1.092	1.126
Sex	−.5.94	.124	22.980	<.001**	.552	.433	.704
Block 1–3							
FAI	−.031	.092	.116	.734	.969	.808	1.162
Age	.103	.008	170.298	<.001**	1.109	1.092	1.126
Sex	−.6.27	.130	23.345	<.001**	.534	.414	.689
School years	−.001	.020	.002	.963	.999	.961	1.039
WST-IQ	−.004	.005	.536	.464	.996	.986	1.007
Block 1–4							
FAI	−.060	.094	.411	.521	.942	.783	1.132
Age	.103	.008	171.525	<.001**	1.109	1.092	1.126
Sex	−.663	.132	25.266	<.001**	.515	.398	.667
School years	−.001	.020	.005	.946	.999	.961	1.038
WST-IQ	−.004	.005	.438	.508	.996	.986	1.007
BDI-II	.013	.008	2.554	.110	1.014	.997	1.030
Block 1–5							
FAI	−.127	.095	1.792	.181	.881	.732	1.061
Age	.080	.009	78.041	<.001**	1.083	1.064	1.103
Sex	−.418	.145	8.253	.004**	.659	.495	.876
School years	.009	.020	.181	.670	1.009	.970	1.049
WST-IQ	.008	.006	1.722	.189	1.008	.996	1.020
BDI-II	.010	.008	1.519	.218	1.010	.994	1.027
NTBV							
F1 Attention	.349	.062	31.198	<.001**	1.417	1.254	1.602
F2 Executive function	.249	.058	18.634	<.001**	1.283	1.146	1.437
F3 Memory	−.226	.071	10.134	.001**	.798	.694	.917
F4 Planning	−.028	.065	.179	.672	.973	.856	1.105
F5 Language	−.153	.069	4.986	.026*	.858	.750	.981
F6 Nonverbal fluency	−.069	.066	1.108	.293	.933	.820	1.062

**p ≤.01, *p ≤.05

The main issue of whether and to what extent the subjective memory performance (FAI) has an explanatory value for mortality in the study population has to be denied due to the non-significant results in the individual model blocks (*p*’s ≥ 0.181). Similarly, depressive symptoms (BDI-II), years of schooling, and verbal intelligence (WST-IQ) cannot be regarded as potential risk factors for mortality. Rather, increasing age (HR 1.083, *p* < 0.001) and male gender (inverted HR 1.517, *p* = 0.004) were found to be relative risk factors. Subsequently, in the 5th model block, four out of the six NTBV dimensions emerged as significant factors with explanatory value; a decreased attention (HR 1.417, *p* < 0.001), a diminished executive function (HR 1.283, *p* < 0.001), a reduced memory (inverted HR 1.253, *p* = 0.001), and a declined language (inverted HR 1.166, *p* = 0.026) can be assumed as relative risks for higher mortality.

## Discussion

The current evidence regarding the impact of cognitive impairment in elderly people on their mortality remains inconclusive. While several studies have suggested that naMCI and aMCI are linked to increased mortality, SCD has not consistently been identified as a significant risk factor [31–34, 37–42]. Nevertheless, some findings report an elevated risk of mortality in individuals diagnosed with SCD compared to cognitively unimpaired controls [35], with variations depending on the cognitive domain affected [36]. In contrast, other investigations have revealed no significant association between cognitive impairment and mortality [3, 43–45].

The inconclusive nature of the findings likely reflects the heterogeneous etiologies underlying subjective and objective cognitive impairment, as well as additional factors influencing patients’ mortality and warrant further research. In this context, we aimed to investigate for the first time whether self-perceived memory performance is associated with 5-year mortality in cognitively impaired patients (SCD, naMCI, aMCI).

Subjective memory function was assessed using the FAI. Results indicated subjective memory ratings decline with increasing cognitive impairment, with significantly lower scores in the aMCI group compared to both SCD (*d* = 0.33) and naMCI (*d* = 0.19). However, no substantial association of deterioration of FAI scoring and increasing age could be observed, except for the SCD group (*r* = 0.18). These findings suggest a reduced self-awareness of memory decline among patients with MCI, aligning with previous studies [18, 29, 30].

The cognitive performance of the patients was assessed using the MMSE, WST-IQ, and VVT 3.0, whereby, as expected, a hierarchical performance gradient of SCD > naMCI > aMCI could be observed.

Neuropsychological functions were examined by using NTBV, containing 24 subtests. A principal component analysis (PCA) suggests a six-factorial solution, the following cognitive domains: attention, executive function—phonematic verbal fluency, memory, planning, language—verbal intelligence, nonverbal fluency; based on the nomenclature of the NTBV domains. These six components imply a reasonable probability of distinguishing SCD and aMCI. The comparison of SCD and naMCI also shows substantial differences in all factors, except the memory dimension, confirming a preserved memory performance in this group. Comparisons between naMCI and aMCI revealed group differences primarily in the memory domain as expected [21, 22].

While 5-year survival rates differed significantly between the diagnostic subgroups—lower survival observed in aMCI (89.9%) compared to SCD (97.6%)—this was likely influenced by the higher age of aMCI patients at testing. Additionally, no substantial disparities were observed in the median age at death across the entire study population or within specific subgroups when compared to the Viennese expectancy of life in 2023 [68].

Regarding these circumstances and the results of the model test via Cox regression, no substantial predictive value can be derived using FAI for estimating mortality in cognitively impaired individuals (HR ≈ 1). It can be hypothesized that the subjective memory may be subject to bias due to social desirability on the one hand and impaired self-perception on the other. However, the covariates age (HR = 1.08) and male gender (inverted HR = 1.52) were considered risk factors.

Also, NTBV dimensions were significantly associated with mortality risk. Declines in Attention (HR = 1.42), Executive function (HR = 1.28), Memory (inverted HR = 1.25), and Language (inverted HR = 1.17) were linked to a higher risk of mortality. This is further substantiated by prior studies, which identified Attention [3, 43–45], Executive functions [43, 44], Memory [3, 44], and Language [44] as key risk domains. Some studies also detected Perceptual speed as an additional factor [43]. Although the exact NTBV dimension structure varies slightly across studies due to methodological differences, the general pattern of findings remains robust and indicates the potential of NTBV as a prognostic instrument.

From a clinical perspective, these findings imply that objective cognitive testing should be prioritized over subjective memory assessments when estimating mortality risk in elderly patients with cognitive impairment.

It should be acknowledged that SCD, aMCI, and naMCI may be based on different underlying pathologies, which in turn can influence mortality outcomes. So does aMCI often reflect underlying Alzheimer’s pathology, where mortality is primarily related to the systemic consequences of progressive neurodegeneration, such as frailty and infection [69]. Also, the so-called VCI is associated with various cardiovascular risk factors that have been shown to increase mortality rates [3, 70–72]. In cases where cognitive symptoms are linked to affective or psychiatric causes, behavioral and metabolic factors may contribute more strongly to mortality risk [73]. In the present study, no distinction was made considering the underlying etiologies or comorbidities, but patients with known medical terms leading to cognitive decline or known pseudo-dementia caused by psychiatric problems became excluded.

However, the severity of depressive symptoms was evaluated via the BDI-II in our study, as those symptoms can be an early sign of neurodegenerative changes and are closely interwoven with subjective and objective cognitive decline, as they may be the cause of it or caused by it [4, 14]. Due to the statistical analyses, only patients with naMCI exhibited slightly elevated levels of depressive symptoms compared to those with SCD, indicating a small effect and depressiveness could not be detected as a potential mortality risk factor referring to Cox-regression.

We also wanted to find out whether there is a correlation between subjectively (FAI) and objectively measured memory performance (NTBV F3). It turned out weak (*r* = −0.16), supporting prior findings that subjective complaints often do not align with neuropsychological tests [5, 6]. It should be considered that SCD may sometimes represent an early manifestation of neurodegenerative processes. In such cases, cognitive test results may still appear unimpaired but nevertheless indicate a decline from a previously higher level of functioning. Only longitudinal follow-up can show whether this decline progresses over time. A closer look at specific neuropsychological subdomains may also provide additional insight, as subtle early changes might already reflect preclinical alterations [9].

It is important to acknowledge certain limitations inherent in this study. Incomplete data were observed for 68 participant protocols for WST-IQ and BDI-II or NTBV (> 20% missing), which had to be excluded from the analyses. Despite the exclusion of individuals with other known conditions affecting cognition, no data were collected on current medication use. As certain drugs may affect both cognitive function and mortality, this represents a potential confounding factor. Furthermore, no cognitively normal control group was included in the study. Instead, age at death was compared with life expectancy data from the general Viennese population. Given the likelihood that this reference group encompasses both cognitively impaired and unimpaired individuals, the validity of this comparison may be constrained. Notwithstanding the slightly higher proportion of female subjects in the study sample, this distribution did not differ significantly from that of the age-matched Austrian population, thereby minimizing the risk of gender-related selection bias.

In summary, this study indicates the limited predictive value of subjective memory for mortality in cognitively impaired individuals. In contrast, declines in specific cognitive domains—especially Attention, Executive Function, Objective Memory, and Language—emerge as risk indicators. These findings underscore the utility of structured neuropsychological testing in identifying at-risk individuals and guiding clinical decision-making.

Due to the retrospective character of the study, further research is required to critically evaluate the observed effects and their actual clinical relevance.

## Data Availability

The data can be obtained upon reasonable request.
